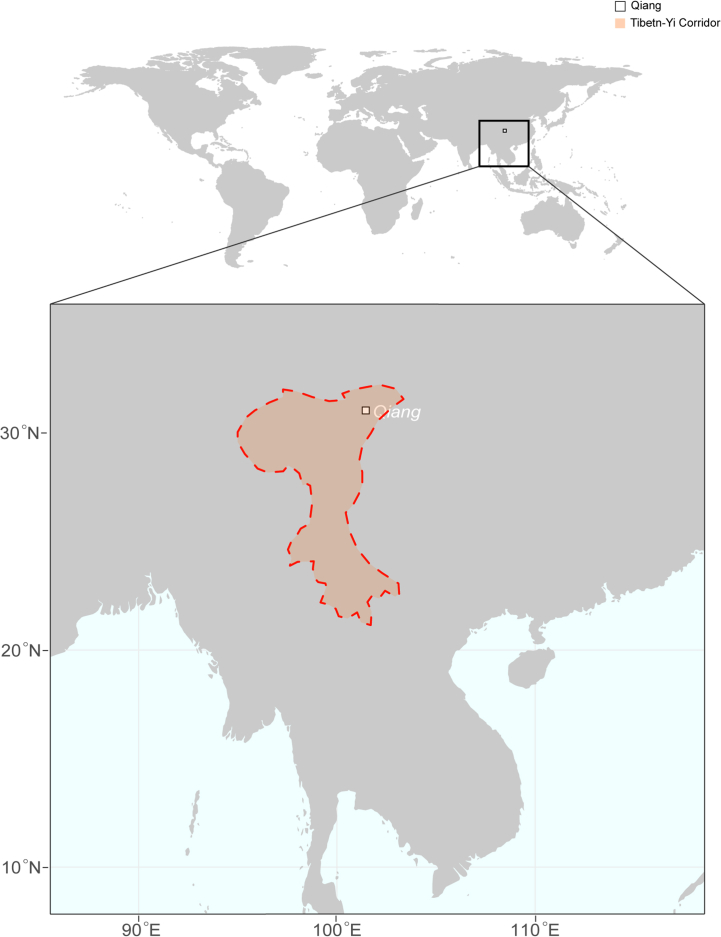# A forensic population database of autosomal STR and X-STR markers in the Qiang ethnic minority of China

**DOI:** 10.1016/j.heliyon.2023.e21823

**Published:** 2023-11-07

**Authors:** Zefei Wang, Mengyuan Song, Qiang Lyu, Jun Ying, Qian Wu, Feng Song, Lanrui Jiang, Xiaowen Wei, Shuangshuang Wang, Fei Wang, Yuxiang Zhou, Xingbo Song, Haibo Luo

**Affiliations:** a Department of Forensic Genetics, West China School of Basic Medical Sciences & Forensic Medicine, Sichuan University, Chengdu, 610041, China; b Department of Laboratory Medicine, West China Hospital, Sichuan University, Chengdu, 610041, China; c Department of Clinical Laboratory, People's Hospital of Beichuan Qiang Autonomous County, Beichuan, 622750, China; d Department of Clinical Laboratory, Santai People's Hospital, Santai, 621100, China; e Department of Clinical Laboratory, Renmin Hospital of Wuhan University, Wuhan, 430060, China

**Keywords:** Population genetics, Short tandem repeat, X chromosome, Autosomal chromosome, Allele frequency, Interpopulation studies

## Abstract

The Qiang ethnic group is one of the oldest ethnic groups in China and is the most active ethnic group among all the populations along the Tibetan-Yi corridor. They have had a profound impact nationally and internationally. The paternal and maternal genetic feature of the Qiang ethnic group has been revealed, leaving the question of the genetic characteristics from autosomes and X chromosome not answered. The aim of this study was to explore the potential of 36 A-STR (Microreader™ 36A ID System) and 19 X-STR (Microreader™ 19X System) for application in the Qiang population and to elucidate their genetic diversity in southwest China. The cumulative probability of exclusion (CPE) for autosomal STRs is 1–1.3814 × 10^−15^ and the mean paternity exclusion chance (MEC) for X-STRs is 1–1.7323 × 10^−6^. Forensic parameters suggest that the STRs analyzed here are well-suited for forensic applications. The results of phylogenetic, interpopulation differentiation, and principal coordinates analysis (PCoA) indicate that the Qiang people have extensive connections with ethnic minorities in China, supporting the view that the Qiang people are the oldest group in the entire Sino-Tibetan language family. The Qiang appeared genetically more associated with most ethnic groups in China, especially the Han. The calculation of random matching probability (RMP) was improved by F_st_ correction of allele frequencies to make RMP more accurate and reasonable. This study can fill in the gaps in the Qiang STR reference database, providing valuable frequency data for forensic applications and evidence for the Qiang's genetic pattern as an important ancestral position in the Sino-Tibetan populations.

## 1 Introduction

The Qiang people, an ancient ethnic group, live mainly in the mountains near the Min River. The name “Qiang” first appeared on oracle bones to record the rich interactions and wars between the Han and Qiang people during the Shang Dynasty [1]. The Qiang people have been migrating throughout their long history, ranging from Central Asia in the west to Shandong in the east, and from the Brahmaputra Rivers in the southwest to the Tibetan-Yi Corridor in the south [2]. The Tibetan-Yi corridor is a region rich in cultural heritage and demographic diversity due to a variety of factors, including geographic and historic reasons [3]. The Qiang people are the most active ethnic group among all the populations along the Tibetan-Yi corridor. They have had a profound impact on its neighboring ethnic groups, which is reflected in the production of stone tools, pottery, bronze and other artefactual tools, ornamental features, habitation, clothing, and hair ornaments [4]. Their place of residence is also referred to as the Tibetan-Qiang-Yi corridor [4].

The Qiang have close ties with more than a dozen of ethnic groups in the southwest of China, some of which are believed to share a common ancestor with the Qiang, while others have received a considerable impact from the Qiang. During the Western Jin Dynasty, conflicts between the Qiang, Han, and Hu people were frequent due to the government's weak control over the Min River region [1]. By the Tang dynasty, with the rise of Tubo's power, the Qiang region became a territory long contested by the Tang and Tubo. Alongside the warfare, the Qiang embraced a great deal of Tibetan culture. In the late Ming Dynasty, many Han Chinese began to move into Qiang territory, bringing with them Han production techniques. In the mid-seventeenth century, the Qing court strengthened its centralisation and the Qiang region gradually entered a feudal landlord economy, and exchanges between the Qiang and Han peoples were further strengthened.

Beichuan Qiang Autonomous County locates in the western part of Mianyang City and the northwestern part of the Tibetan-Yi corridor. Beichuan Qiang Autonomous County is the only Qiang autonomous county in China, located in the northwest part of the Sichuan Basin. It went through a big earthquake in 2008, which was known as “the Wenchuan earthquake”. According to the 7th official population consensus [5], the total population of Beichuan Qiang Autonomous County is about 230,000, of which 86,194 are Qiang, accounting for 37.3 % of the total population [1].

Current research on the Qiang ethnic group mainly focuses on history and culture, while studies on the genetic characteristics of the Qiang population have mainly focused on the Y chromosome [6]. Short tandem repeat (STR) polymorphisms play an important role in forensic genetic identification and the study of population evolutionary history [7]. Autosomal short tandem repeats (A-STRs) are the most commonly used STR markers in personal forensic identification and kinship testing. China is a multi-ethnic country with a large population. The differences in gene frequencies among the various ethnic groups and the data generated by the large population are a constant challenge for forensic applications. Random matching probability (RMP) is always calculated using the allele frequencies of combinations of multiple populations within a larger group such as a province or even a country. A broad combination may contain multiple populations with a more even distribution of gene frequencies. The calculated RMP and likelihood ratio (LR) using non-matched allele frequency reference may be unfavorable to the suspect.

To further trace the Qiang tribe and provide a reference for forensic applications, we constructed an STR database of the Qiang including A-STRs and X-STRs. It is beneficial for forensic practice, as well as studying the genetic relationship and origin between the Sichuan Qiang population and other Chinese reference populations. X-STRs can complement the analysis of A-STRs, e.g. in cases of paternity where the information obtained from standard autosomal markers is uncertain, and in cases of incest where possible related fathers are distinguished [8].

## 2 Materials and METHODS

### 2.1 Ethical requirements and sample collection

Blood samples were collected from 421 healthy, unrelated, and adult voluntary citizens (333 male and 88 female) living in Beichuan Qiang Autonomous County for three generations. Its approximate location on the map and the extent of the Tibetan-Yi Corridor are shown in Supplementary Fig. 1. A-STR and X-STR typing were performed using 205 male samples together, and X-STR typing was carried out in an additional cohort of 128 male and 88 female samples. This study was approved by the Biomedical Research Ethics Committee of West China Hospital, Sichuan University (2022882). All blood samples were collected following the international ethics rules: signed consent from all donors and further securing of anonymity were provided. According to the decision of the local ethics committee, all DNA samples isolated from these blood samples were used for research purposes only.

### 2.2 DNA extraction, PCR amplification, and genotyping

DNA of all samples was extracted using the Chelex-100 method [9]. The supernatant was aspirated after centrifugation at 12,000 rpm for quantification. DNA was quantified with NanoDrop 2000c (Thermo Fisher Scientific, Waltham, MA, USA) according to the manufacturer's instructions. DNA samples were diluted to the concentration of 1 ng/μl for PCR according to the recommended protocol for the Microreader™ 36A ID System (Beijing Micro-reader Genetics, Beijing, China) and Microreader™ 19X System (Beijing Micro-reader Genetics, Beijing, China) PCR Amplification Kit. The Microreader™ 36A ID System uses a 6-color fluorescent marker with multiplex amplification to detect 37 STR motifs (i.e., TH01, D5S818, D21S11, D18S51, D6S1043, D15S659, D6S477, D3S1358, D13S317, D7S820, D16S539, CSF1PO, Penta D, D8S1132, D7S3048, D2S441, vWA, D8S1179, TPOX, Penta E, D14S608, D4S2366, D3S3045, D19S433, D22S1045, D2S1338, FGA, D5S2500, D10S1435, D18S535, D1S1656, D12S391, D10S1248, SE33, D19S253, D11S2368, DYS391), one sex locus Amelogenin and one Y Indel locus (Rs2032678). The Microreader™ 19X System uses a 5-color fluorescent marker with multiplex amplification to detect Amelogenin and 19 STR motifs (i.e., DXS6795, DXS6803, DXS6807, DXS9907, DXS7423, GATA172D05, DXS101, DXS9902, DXS7133, DXS6810, GATA31E08, DXS6800, DXS981, DXS10162, DXS6809, GATA165B12, DXS10079, DXS10135, HPRTB).

1 ng template was added to 10 μl of Master Mix III and 5 μl Primer Mix, and then nuclease-free water was added up to 25 μl for Microreader™ 36A ID System. Thermal cycler conditions included an initial incubation for 5 min at 95 °C; 28 cycles of denaturation for 20 s at 94 °C, annealing/extension for 90s at 59 °C and final extension for 60 min at 60 °C; and a final hold at 4 °C. Microreader™ 19X System total reaction volume was 25 μl, including 10 μl of Microreader™ 2.5 × Buffer, 5 μl of Microreader™ 19 X-STR 5 × Primer Mix, 0.1 μl Taq, and 0.5 μl ng of template DNA, and then nuclease-free water was added up to 25 μl. The thermal cycling parameters were set as follows: preincubation at 95 °C for 5 min; 29 cycles of denaturation at 94 °C for 30 s, annealing at 59 °C for 60 s, and extension at 72 °C for 60 s; extension at 60 °C for 30 min, finally holding at 25 °C until the amplification products were removed from the PCR machine.

An Applied Biosystems 3500 Genetic Analyzer (Thermo Fisher Scientific, Waltham, MA, USA) was used for all amplification products, using a 36 cm capillary array and POP-4 polymer. 1 μl of PCR products was added to a 9 μl mixture of Hi-Di formamide and a microreader size standard. STR profiles were analyzed by GeneMapper™ ID-X v1.5 software (Appied Biosystems Foster City, CA, USA). Allele lengths were determined by comparing the sample PCR fragments with the allelic ladder provided with the kit.

### 2.3 Reference database

The STR data of Qiang was compared with the data of ethnic minorities in China derived from published sources as summarized in Table 1. Database I contains autosomal STR data. PCoA, heat map, and structural analysis were performed using 15 overlapping A-STRs. Both Database II and Database III are X-STR data. The difference is that the former has more populations but 7 overlapping X-STRs and the latter consists of fewer populations but uses all 19 overlapping X-STRs. Both were subjected to PCoA and heat map analysis.

**Table 1:** Datasets for various analyses in this study.

Dataset	Analysis	Minorities	Data
Dataset I	PCoA, heatmap, Structure	Hui, Tibetan and Uygur [10]	A-STRs
		Mongol, Kyrgyz and Uzbek [11]	
		Yi [12]	
		Han [13]	
		Du Long and Lisu [14]	
		Hui [15]	
		Xibe [16]	
		Nakhi and Yi [17]	
		Han [18]	
		Uygur [19]	
		Mongolian [20]	
		Myanmar [21]	
		Han, Nu and Tibetan [22]	
Dataset II	PCoA, heatmap	Han and Hui [23]	X-STRs
		Han [24]	
		Mongolian [25]	
		Han [26]	
		Miao [27]	
		Daur and Oroqen [28]	
		Uygur and Tibetan [29]	
		Zhuang and Mulao [30]	
		Miao [31]	
		Tibetan [32]	
		Yi [33]	
		Kyrgyz and Han [34]	
Dataset III	PCoA, heatmap	Korean [35]	X-STRs
		Han [36]	
		Gelao [36]	
		Miao [36]	
		Kazakh [37]	
		Tujia [37]	
		Han [38]	

### 2.4 Statistical analysis

Statistical parameters of forensic interest include power of discrimination (PD), matching probability (MP), observed (HO) and expected (HE) heterozygosities, polymorphism information content (PIC), typical paternity index (TPI), power of exclusion (PE) and allele frequencies were determined using modified STRAF 1.0.5 [39] and ChrX-STR.org 2.0 [40]. Hardy–Weinberg equilibrium (HWE) and pair-wise linkage disequilibrium (LD) in female samples were calculated by the software of Arlequin 3.5.2 [41]. We used the Principal Coordinate Analysis function in GenAlEx 6.503 [42] to perform a Principal Coordinate Analysis (PCoA). The ridge plots were achieved by *ggridges* and *ggplot2* packages in R. The heat map is implemented by the *readxl*, *gplots*, packages in R. The geographic map was drawn by R packages *ggplot2*, *maps*, *sf*, *rnaturalearth,* and *rnaturalearthdata*.

We used 196 Qiang individuals (9 individuals were removed that contained off-ladder (OL) alleles absent in the Han Chinese database) at 21 autosomal loci (CSF1PO; D10S1248; D12S391; D13S317; D16S539; D18S51; D19S433; D21S11; D2S1338; D2S441; D3S1358; D5S818; D6S1043; D7S820; D8S1179; FGA; PentaD; PentaE; TH01; TPOX; vWA) to analyze the effect of F_st_ correction on RMP. The exact test for population differentiation (F_st_) was carried out by Arlequin3.5.2 software [41]. The RMP calculation was performed by EuroForMix_3.0.3 [43]. It fills in the F_st_-correction value in the settings and recalculates the corrected RMP. Afterwards, the individual RMP values were compared and the log10 of the ratio was calculated. The RMP was corrected using the correction formula from David J. Balding and Richard A. Nichols [44] (in formula F represent F_st_), the derivation of which is described in detail in the text. The two observed alleles were denoted by A and B, and their proportions in the database population were represented by p_A_ and p_B_. Then the probability that a suspect has these alleles given that the offender has them is.

For homozygotes, RMP is defined as:

(1) $$\begin{array}{c} \frac{\left[2F+\left(1-F\right)p_{A}\right]\left[3F+\left(1-F\right)p_{A}\right]}{\left(1+F\right)\left(1+2F\right)} \end{array}$$

For heterozygotes, RMP is calculated as:

(2) $$\begin{array}{c} \frac{2\left[F+\left(1-F\right)p_{A}\right]\left[F+\left(1-F\right)p_{B}\right]}{\left(1+F\right)\left(1+2F\right)} \end{array}$$

For structure analysis for autosomal STR, the software Structure v2.3.4 [45] was used with the under the following parameters: The evaluated number of clusters (K) ranged from 2 to 8; for each K, 10 independent runs were performed. We utilized a 100,000-iteration burn-in period followed by 50,000 iterations. Using the Structure Harvester, we assessed the number of genetic groups (K) that best fit the data. Finally, the CLUMPP_Windows.1.1.2 [46] program was used to output a mean of the permuted matrices across replicates for each K. Based on the best K, we estimated and plotted the percentage of admixture in each population with distruct1.1 [47].

## 3 Results

### 3.1 Allelic frequencies and forensic parameters of A-STRs

The allele frequencies and forensic parameters of the 36 autosomal STR loci are listed in Supplementary Table 1. A total of 424 alleles were observed in the studied population, with corresponding allele frequencies from 0.0025 to 0.4015. There were no allele frequencies greater than 0.5, indicating that these motifs are well heterozygous in the Qiang population and that the allele frequencies are evenly distributed. There was no significant deviation from HWE after applying the Bonferroni correction, except for locus D6S477. The deviation may be due to undersized populations, inbreeding, population substructure, or selection. Another important factor may be the small sample size, and increasing the sample size may make it no longer deviate from the HWE. P-values for the Linkage disequilibrium test between all A-STRs are shown in Supplementary Table 2, with no significant linkage between any of the A-STRs.

PIC was in the range of 0.584 (TPOX) to 0.948 (SE33), and the most discriminating marker was SE33 with a PD value of 0.989. The observed heterozygosity varied from 0.567 (TPOX) to 0.951 (SE33), and the PE ranged from 0.253 (TPOX) to 0.900 (SE33). The CPD, CPE, and CPM were 1–7.8531 × 10^−44^, 1–1.3814 × 10^−15^, and 7.85314 × 10^−44^, respectively. This indicated that the 36 autosomal STR loci were highly polymorphic and appropriate for individual identification and paternity testing in the studied population.

### 3.2 Improvement of RMP calculation by F_st_ correction

Using a non-cognate database calculation in the RMP calculation can be detrimental to the suspect, so it is more appropriate to use F_st_ correction. We tested the impact of using different reference frequencies on RMP calculations. First, we used the Sichuan Qiang allele frequency database (generated in this study) and Sichuan Han allele frequency databases, for the RMP calculation of the Qiang individuals. The selected Sichuan Han reference dataset was from a study of 2793 unrelated individuals. In China, the Han Chinese are the dominant population and its frequency database is commonly used in forensic practice. We used 196 Qiang individuals (9 individuals were removed that contained off-ladder (OL) alleles absent in the Han Chinese database) of the Qiang ethnic group to calculate the RMP using the allele frequencies of their population and that of the Sichuan Han ethnic group. Calculated using the formula in section 2.4.

**Fig. 1:**
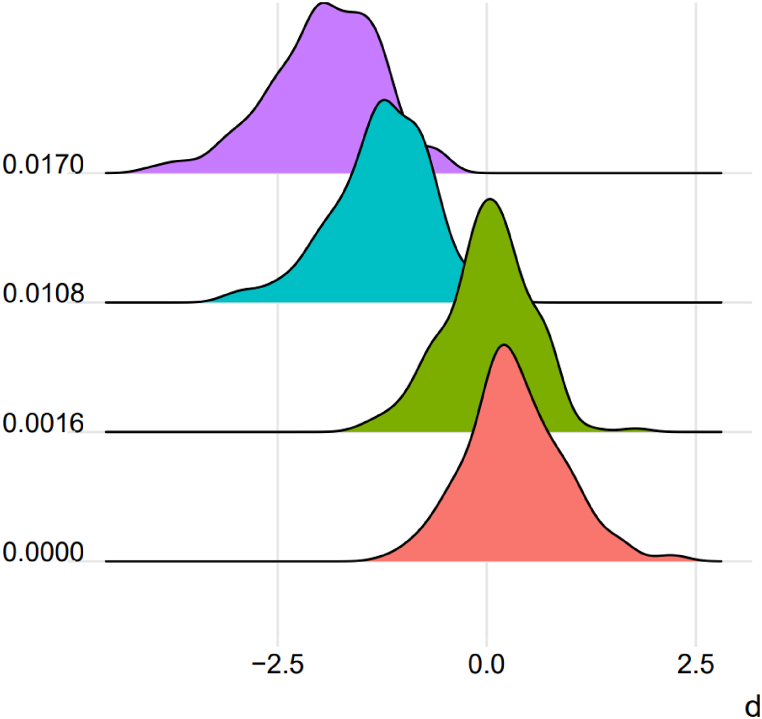
Distribution of d values after correction using different F_st_. Colors indicate different F_st_ adjustment.

Then a parameter d was calculated, corresponding to log10 of the ratio between RMPs obtained from the A-STR profiles of each individual from the Qiang population when using cognate (Qiang) and non-cognate (Han) allele frequencies [48]. The results are displayed in Table 2 and Fig. 1. Values of d >0 indicate an overestimation of the weight of the evidence when using the non-cognate databases for calculations.

**Table 2:** The results of d values obtained by applying different F_st_ adjustment when using Sichuan Han allele frequencies for RMP calculation. F_st_ values were recommended by Xuan Dai et al. for the Chinese populations [49].

Table 2. Distribution of values.F	d >0	d >0.5	d.>1	d >2	Mean d	±SD
0	74.49 %	35.20 %	12.24 %	1.02 %	0.3309	0.5848
0.0016	54.08 %	18.37 %	01.02 %	0.00 %	0.0102	0.5102
0.0108	1.02 %	00.51 %	0.00 %	0.00 %	−1.1970	0.5878
0.0170	00.51 %	00.51 %	0.00 %	0.00 %	−1.8538	0.6630

It can be seen that without F_st_ correction (F is set as zero), there is a slight overestimation of the weight of evidence. Applying the F_st_ correction of 0.0016 is more accurate when using the Sichuan Han database for RMP calculations, with an average d of 0.0102. However, the weight of the evidence in more than half of the cases was still overestimated, which would be magnified by the hypothesis that the suspect was related to the offender [44]. It would be more appropriate to use 0.0108, for the risk of non-conservative estimations to become almost negligible, with only 1.02 % of individuals overestimating the weight of the evidence and an average RMP one order of magnitude higher, which is acceptable in practice. For the Qiang, the use of 0.0016 is appropriate in the general case. However, when it comes to family exclusion, it is feasible to add sex chromosome markers or use a larger F_st_. When a population has more than one recommended F_st_, it is most appropriate to select the F_st_ that is within the smallest range that can contain cognate and non-cognate databases. The F_st_ used for the correction cannot be applied to all cases, so the use of a cognate database is necessary.

### 3.3 Population affinity revealed by A-STRs

The pairwise F_st_ values and corresponding p-values between Qiang and other reference populations (listed in Table 1) are shown in Supplementary Table 3 (using overlapping 15 A-STRs). The largest F_st_ value (0.04436) was observed between the Fujian, She and Yunnan, Du long ethnic groups, whereas the smallest F_st_ value (−0.0003) was found between Mongolians from Xinjiang and Inner Mongolia. F_st_ values are larger between ethnic groups in Yunnan and other ethnic groups due to their ethnic diversity and smaller sampling. In general, the F_st_ value increases with geographic distance.

**Fig. 2:**
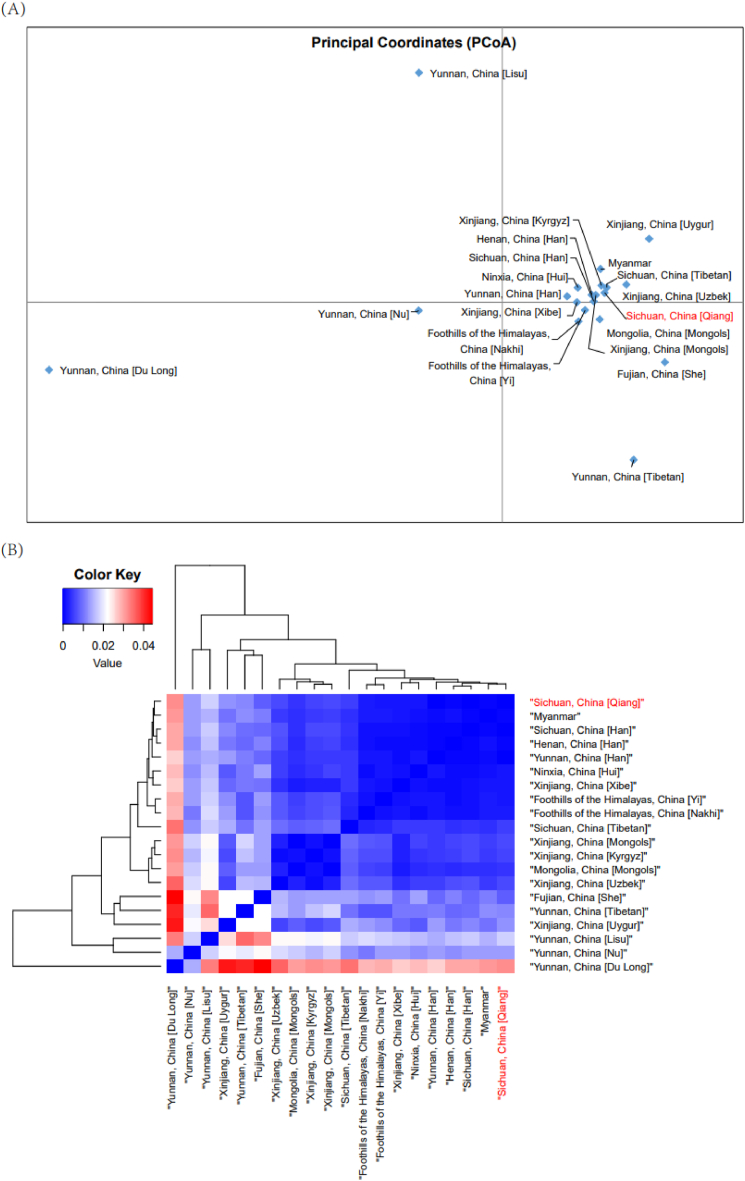
Genetic relationships estimated by genetic distances of autosomes (A-STRs) between Qiang (red) and the reference database are represented in principal coordinates analysis (PCoA) (A) and heatmap (B). In general, there is a tendency for different ethnic groups to cluster by region and ethnicity.

The results of the PCoA are shown in Fig. 2A. It graphically represents the genetic relationships between the studied population and the other 19 reference populations. As can be seen from Fig. 2A, Yunnan minority groups are distributed on the edge of the figure. Most of the ethnic group cluster on the right side of the figure, which suggests a relatively low degree of differentiation. In general, there is a tendency for different ethnic groups to cluster by region and ethnicity. The Sichuan Qiang and Sichuan Tibetans are close together in the upper right quadrant of the figure. In the Y chromosome haplogroup, we studied before [50], both Qiang and Tibetan have a portion of haplogroup D. Both results illustrate the close relationship between the Qiang and Tibetans. It supports the view that the Qiang are the ancestors of both Tibetans and Han Chinese [50]. Similarly, the heatmap (Fig. 2B) was constructed based on the genetic distance of F_st_. The Qiang are clustered with the ethnic groups in the areas around the Tibetan-Yi corridor and with the Han Chinese in various regions, which illustrates the close relationship between the Qiang and the ethnic groups in the Tibetan-Yi corridor and the extensive contact with the Han Chinese. The ethnic groups of Xinjiang and Mongolia are clustered together, while the rest of the ethnic groups are scattered in other locations, with the Dulong of Yunnan located in a separate group, a small society of only 4000 people with little interaction with modern society [51].

**Fig. 3:**
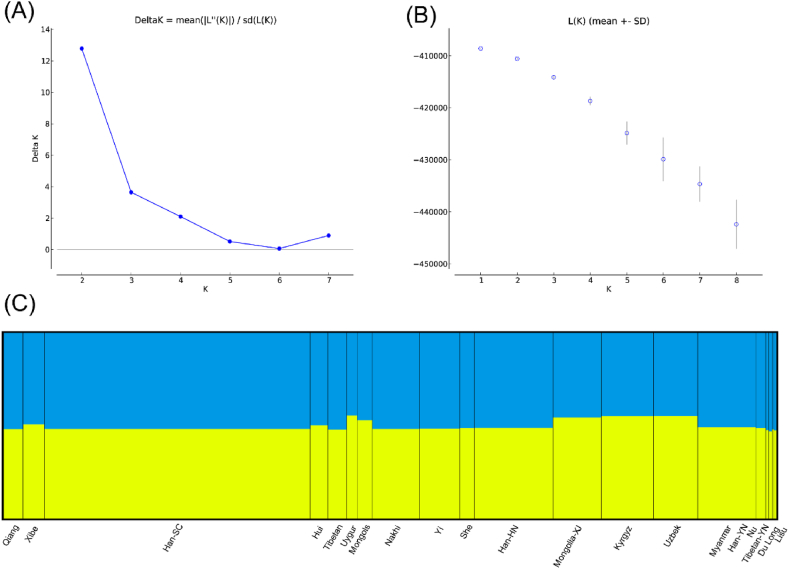
Results of STRUCTURE analysis. (A) Illustrates maximum of delta K (B) Illustrates maximum of L(K) (C) Bar plot representing structure analysis of Qiang in comparison to 19 other populations based on 15 autosomal STRs when k = 2.

STRUCTURE analysis was performed using Structure v2.3.4 software to genotype 15 STRs from the Sichuan Qiang and other published ethnic groups. Each K = 2–8 for 10 runs; then, the optimal K was selected using Structure Harvester and the results of the calculations are displayed in Fig. 3A,B, showing that K = 2 is the most appropriate. However, we do not find a significant difference in the structure plot for K = 2 (Fig. 3C). In Fig. 3B we can see that K = 1 has the highest L(K), which means that K = 1 should be more appropriate. Structure Harvester recommends the best K = 2 as its algorithm does not generally recommend K = 1 [46]. These populations tend to have an identical ancestor, which may be due to the geographical proximity of the selected populations. The populations we have selected are all from the Tibetan-Yi corridor or western China and all belong to the Sino-Tibetan language family, of which Qiang, Tibetan, Yi, Dulong and Burmese all belong to the Tibetan-Burmese branch, so it is reasonable to obtain this result.

### 3.4 Allelic frequencies and forensic parameters of X-STRs

A total of 333 male and 88 female individuals from the Sichuan Qiang group were analyzed. Allele frequencies for males, females, and pooled are presented in Supplementary Table 4, Supplementary Table 5 and Supplementary Table 6 respectively. The F_st_ and corresponding p values of 19 X-STRs between females and males in Sichuan Qiang were presented in Supplementary Table 7. No gender differentiation is identified after Bonferroni correction (p = 0.05/19 = 0.0026). Lower P values were observed for DXS6800, which may be due to the low polymorphism of DXS6800 and the small number of female samples. A total of 180 alleles were observed at the 19 X-STR loci, and the allele numbers ranged from 5 at DXS7133 and DXS7423 to 22 at DXS10135. The most frequent allele observed was allele 16 at locus DXS6800, with a frequency of 0.8490. Bonferroni-corrected HWE exact test (Supplementary Table 8) (p = 0.05/19 = 0.0026) showed no significant deviation for the 19 X-STR motifs in the female sample except for DXS7423.

In the linkage group test (Supplementary Table 9), after Bonferroni correction, no evidence of detectable linkage disequilibrium was found for the same pair of markers except for DXS10079 and DXS6800 (p = 0.000292). The haplotype distributions of Sichuan Qiang population are presented in Supplementary Table 10. A total of 28 haplotypes of LG DXS10079-DXS6800 are observed in 333 males, and the most common haplotypes are 16–20(0.2583). The difference is that in a study of Han Chinese in Beijing, China [52] no evidence of detectable linkage disequilibrium was found in DXS10079 and DXS6800. The different results obtained by the Sichuan Qiang and the Beijing Han on the linkage of these two STRs may be due to the small size of our sample or to the unique genetic pattern of the Qiang, which needs to be demonstrated by future experiments with larger samples.

Forensic statistical parameters are listed in Supplementary Table 7. We consider these STRs as 18 loci to calculate the parameters. DXS10135, with the highest PIC (0.9097), HD (0.9176), PDF (0.9868) and PDM (0.9158), had the highest polymorphism. The lowest PIC (0.3052), HD (0.3533), PDF (0.5335) and PDM (0.3527) were observed at DXS7133. Interestingly, we found that the polymorphism of DXS6800 was low in many ethnic groups in China, but not in some other countries. For example, the polymorphism information content (PIC) is only 0.2633 and 0.2651 [38] in the Qiang of Sichuan and the Han of Yunnan, but is high in Germany (0.7314) [53] and Austria (0.7322) [54]. The combined PDF and PDM are 2.0622 × 10^−17^ and 1–9.9450 × 10^−11^, respectively. The combined mean paternity exclusion chance calculated by the formula of MEC_Krüger_(mean paternity exclusion chance), MEC_Kishida_, MEC_Desmarais_, and MEC_Desmarais Duo_ are 1–4.7542 × 10^−7^, 1–7.2687 × 10^−11^, 1–7.3127 × 10–11, and 1–9.2682 × 10^−8^, respectively. The forensic parameters described above indicate that the 19 X-STRs in the Microreader™ 19X PCR amplification kit are highly polymorphic and informative in the Sichuan Qiang population and can be used as a powerful tool for forensic complex kinship identification.

### 3.5 Population affinity revealed by X-STRs

Current research on X-STRs has focused on the analysis of linkage groups, and such linkage-free X-STR kits are less commonly used. Therefore, to explore the genetic homogeneity and heterozygosity of different ethnic groups in X-STR, two databases were chosen for comparison, the first using the Sichuan Qiang and 15 other ethnic groups using overlapping 7 X-STRs, the second group using the Sichuan Qiang and 7 other ethnic groups using overlapping 19 X-STRs.

In the first group we calculated the F_st_ genetic distance between the Sichuan Qiang and 15 other ethnic groups using overlapping 7 X-STRs. The pairwise F_st_ values are listed in Supplementary Table 11. The closest genetic distance (F_st_ genetic distance: 0.002) appeared between Sichuan Qiang and Guizhou Miao and between Sichuan Qiang and Southern Han. Meanwhile, the Qiang also possessed a close genetic distance (F_st_ < 0.05) from the Heilongjiang Daur, Guizhou Han, Sichuan Yi, and Sichuan Han.

**Fig. 4:**
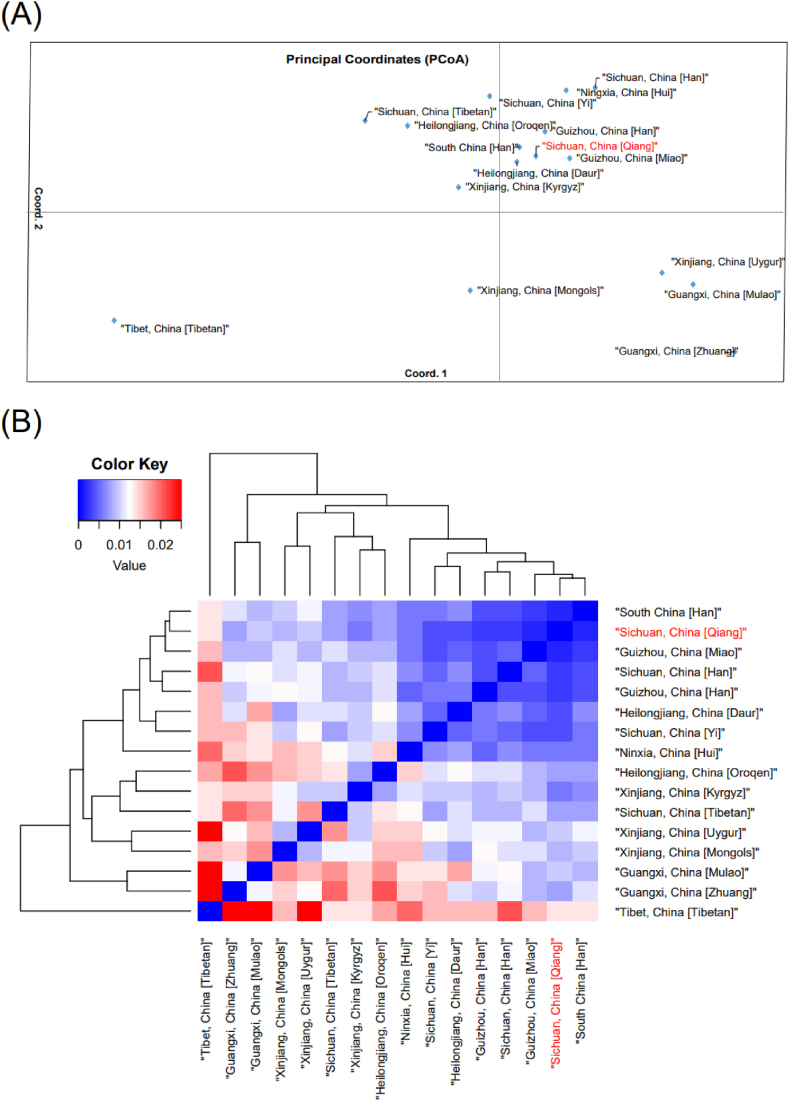
Genetic relationships estimated by genetic distances of X-STRs between Qiang (red) and the reference database containing 15 ethnic groups are represented in principal coordinates analysis (PCoA) (A) and heatmap (B).

PCoA is shown in Fig. 4A, with most ethnic groups showing clustering by geographic location. Guangxi Mulao and Guangxi Zhuang appear in the lower right corner, which is far from Sichuan Qiang population. The Xinjiang minority appears in the middle and lower part of the figure, of which Kyrgyz ethnic group has the most genetic affinity with Sichuan Qiang. Sichuan and Guizhou minorities are clustered surrounding Sichuan Qiang population. Tibetans in Tibet are alone in the lower left, quite separate from Qiang population. The Qiang and the southern Han Chinese are located in the center of the cluster above. Mansha Jia et al. [55] revealed the genetic homology of Oroqen, Mongolian, Kirk, and Uyghur, as evidenced by our study. Heatmap (Fig. 4B) were constructed according to F_st_ genetic distance. The different ethnic groups are distributed approximately according to geographic location, with the Qiang clustering with the south-western Chinese ethnic groups. The Heilongjiang Daur are closer to the south-western ethnic groups. A similar structure was found in previous studies of the autosomes of the Daur ethnic groups, suggesting that the Daur are genetically more closely related to the western Chinese groups. The ethnic groups of Xinjiang cluster together with the Tibetans of Sichuan, and the ethnic groups of Guangxi cluster with each other. The genetic relationship between Tibetans and Han Chinese is different from A-STR, where Qiang are closely genetically related to Tibetans, but in X-STR the Qiang are distantly genetically related to both Tibetans in Sichuan and Tibetans in Tibet.

**Fig. 5:**
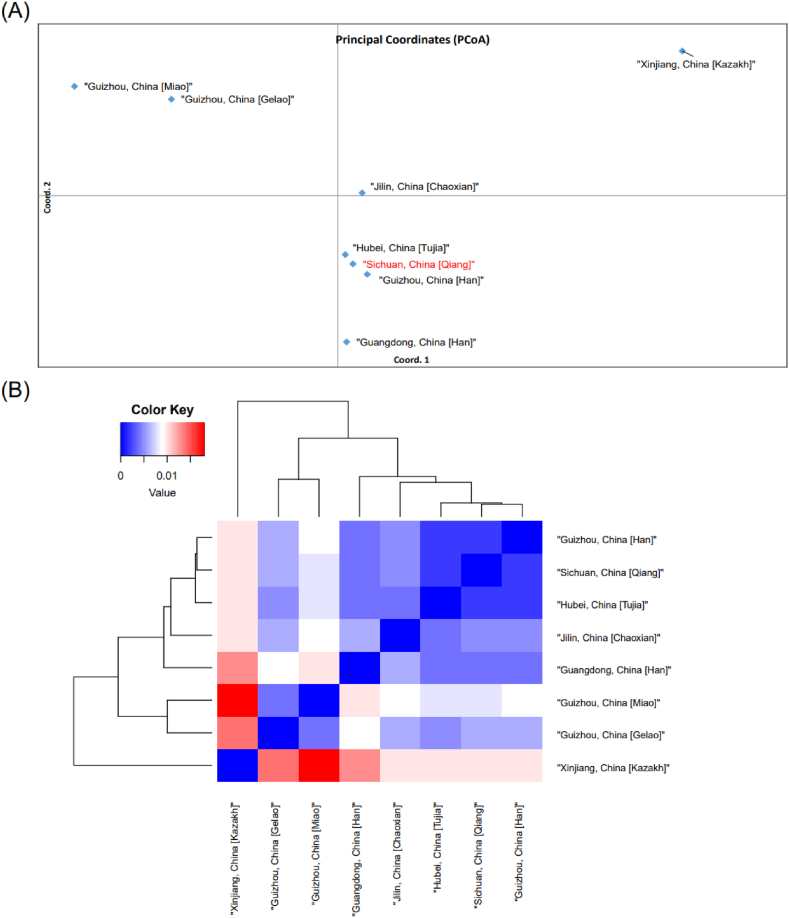
Genetic relationships estimated by genetic distances of X-STRs between Qiang (red) and the reference database containing 7 ethnic groups are represented in and principal coordinates analysis (PCoA) (A) and heatmap (B).

The second group is similar to the first group, the pairwise F_st_ values are listed in Supplementary Table 12. The closest genetic distance (F_st_ genetic distance: 0.002) appeared between Sichuan Qiang and Guizhou Han and between Sichuan Qiang and Hubei Tujia. In the heatmap (Fig. 5B) the Qiang, Tujia and Han form a cluster, the two Guizhou minorities are clustered together and the Kazakhs are in a separate group. PCoA (Fig. 5A) also shows similar results to heatmap. Consistent with previous analyses, the Qiang are closely related to the Han. The Tujia are closely related to the Han in many studies, so the Tujia and Qiang are also clustered together.

In general, Sichuan Qiang showed a genetic affinity with Sichuan Han and extensive genetic heterogeneity with Mongols and Uygur. At the same time, the Sichuan Qiang are genetically homogeneous with most ethnic groups in southwestern China. This is consistent with our expectations and demonstrates that the Qiang has been continuously developing and differentiating since about 1000 BCE [1], with extensive and profound influence on the history and ethnic formation of the surrounding region. Interestingly, Qiang showed a genetic affinity with Tibetans in A-STRs but was not seen in X-STRs. Different results based on the analysis of different chromosomes are also found in other ethnic groups [56,57]. However, it is also possible that this is due to the low number of overlapping X-STRs used, and more Tibetan data are needed to confSequencing of aDNA pirm this phenomenon.

## 4 Discussion

We have typed A-STR for 205 samples and X-STR for 421 samples from the Sichuan Qiang ethnic group and provided allele frequencies for the Sichuan Qiang ethnic group, which will provide a basis for application and ethnic analysis in forensic cases. Of all STRs, we found two (D6S477, DXS6800) that failed HWE after Bonferroni correction, in all cases because the homozygote exceeded expectations. DXS6800 also has a low PIC (0.2634) and a gene frequency of 0.8529 for allele 16. A similar situation is seen in many Chinese ethnic groups, but has a high PIC in Europe and Oceania, suggesting that this locus may not be suitable for forensic applications in the Chinese region.

In practice, DNA analysts generally place more emphasis on genetic profile matches, and the introduction of RMP and LR allows forensic scientists to assess the strength of evidence. When a suspect is from a small and isolated subpopulation, the population allele frequency should be used preferentially to calculate the RMP. if appropriate data are lacking, then the use of F_st_ values corrected for other populations or allele frequencies containing combinations of multiple populations should be applied. If not calibrated, the weight of evidence will be overestimated. For Sichuan Qiang, the use of 0.0016 for F_st_ adjustment is closest to the homology database results, but does not mitigate the overestimation of the evidence in some special cases such as when the suspect is a sibling of the offender. Using 0.0108 mitigates the overestimation of the evidence, but is somewhat overly conservative and underestimates the strength of the evidence across the board. Therefore, the use of a cognate database is more accurate and comprehensive for the estimation of the weight of evidence.

For population genetic comparisons of Qiang and other ethnic groups, our genetic results show its historical complexity, which has a non-negligible role in the evolution of many ethnic groups. In the A-STRs, the Qiang show a wide range of close ties with the populations of the Tibetan-Yi corridor. For X-STRs, the Sichuan Qiang are genetically homogeneous with most ethnic groups in southwestern China. All STR markers showed gene flow between the Qiang and Han individuals, which is consistent with previous studies of the Y chromosome. Song et al. [50] and Wu et al. [6] analyzed the structure of the Qiang on the Y chromosome in terms of the Y-STR and Y haplogroup of the Qiang, both indicating clear gene flow between the Han and the Qiang. The Di-Qiang tribe is an ancient name for both the Di and Qiang tribes, at present the Di are integrated into the Han. A number of studies of ancient DNA of the Di-Qiang people have found that the Di-Qiang contributed to the Han STR gene pool [58], as well as gene flow to the Xinjiang region and the Hexi Corridor [59]. In maternal inheritance, the results of the Qiang mtDNA study show similar results to those of the paternal lineage [60], and the diversity of the Qiang population is also mainly contributed by haplogroups prevalent in North Asia. The difference is that genetic differentiation with Tibetans is shown on the X-STR, which is different from the A-STR and Y-STR, but there are fewer overlapping loci for comparison, so using the same kit for Tibetans would help to further investigate this phenomenon.

## 5 Conclusion

We have typed the 55 STRs contained in the Microreader™ 36A ID System and Microreader™ 19X System in 481 samples from Sichuan Qiang and provide allele frequencies and forensic parameters for the Qiang population in Sichuan, which will provide valuable assistance in the personal identification and paternity testing. Improving the calculation of RMP by F_st_ correction of allele frequencies can make RMP more accurate and reasonable, if non-cognate database was used for RMP calculation. To better understand the genetic structure and inter-population relationships, different STR combinations were applied in the genetic distance calculations to estimate the distant relationships between ethnic groups. Taken together, these results suggest that the Qiang are closely related to the ethnic groups in the Tibetan-Yi corridor and have few genetic differences from the Han Chinese. Our genetic results show its historical complexity, which has a non-negligible role in the evolution of many ethnic groups. The cultural and biological characteristics of this ethnic group are important for population diversity estimation, and further research on it and surrounding population is needed.

## Data availability statement

Data included in article/supplementary material/referenced in article.

## Fundings

This study was supported by grants from the 10.13039/501100001809National Natural Science Foundation of China (81772030, 81672096, 82202614). The funders had no role in study design, data collection and analysis, decision to publish, or preparation of the manuscript.

## CRediT authorship contribution statement

**Zefei Wang:** Data curation, Formal analysis, Writing – original draft. **Mengyuan Song:** Data curation, Investigation, Writing – review & editing. **Qiang Lyu:** Data curation, Formal analysis. **Jun Ying:** Data curation, Formal analysis. **Qian Wu:** Data curation, Formal analysis. **Feng Song:** Conceptualization, Supervision. **Lanrui Jiang:** Data curation, Formal analysis. **Xiaowen Wei:** Software. **Shuangshuang Wang:** Conceptualization, Supervision. **Fei Wang:** Software. **Yuxiang Zhou:** Software. **Xingbo Song:** Conceptualization, Funding acquisition, Supervision, Writing – review & editing. **Haibo Luo:** Conceptualization, Funding acquisition, Supervision, Writing – review & editing.

## Declaration of competing interest

The authors declare the following financial interests/personal relationships which may be considered as potential competing interests:Haibo Luo reports financial support was provided by 10.13039/501100001809National Natural Science Foundation of China. Xingbo Song reports financial support was provided by 10.13039/501100001809National Natural Science Foundation of China. Mengyuan Song reports financial support was provided by 10.13039/501100001809National Natural Science Foundation of China.

## Figures and Tables

**figs1:**